# Electron mobility in semi-metal HgCdTe quantum wells: dependence on the well width

**DOI:** 10.1186/s40064-016-1715-6

**Published:** 2016-01-26

**Authors:** E. O. Melezhik, J. V. Gumenjuk-Sichevska, F. F. Sizov

**Affiliations:** Institute for Semiconductor Physics, National Academy of Sciences of Ukraine, pr. Nauki 41, Kiev, 03028 Ukraine

**Keywords:** HgTe, Mobility, Boltzmann transport equation, Inelastic scattering, Screening

## Abstract

Energy spectra, carrier concentration and electron mobility are numerically modeled in intrinsic and n-type semi-metal HgCdTe quantum wells at T = 77 K. We present results for the electron mobility calculated in a model incorporating electron scattering on longitudinal optical phonons, charged impurities, and holes, and including the 2D electron gas screening for all mentioned scattering mechanisms. Inelasticity of electron–phonon scattering is treated by means of a direct iterative solution of Boltzmann transport equation. Comparison with the experimental data at liquid helium temperature is provided.

## Background

Nowadays the task of creation of fast and sensitive detectors in the THz spectral range is important for various areas such as medicine, security, and aerospace. Among other detector types, semiconductor bolometric detectors allow one to combine high sensitivity, operation speed, and device compactness. Mercury–cadmium–telluride (MCT) heterostructures are promising materials for the implementation of such type of detectors. Hg_1−x_Cd_x_Te quantum wells (QWs) exhibit high electron mobilities and concentrations, even at liquid nitrogen temperatures. In such QWs, depending on the composition *x* and quantum well width *L,* a semi-metallic or semiconducting state can be realized (Olshanetsky et al. 2014). A semi-metallic state is characterized by much higher conduction electron concentration at liquid nitrogen temperatures (Melezhik et al. 2014). Therefore, comparing with undoped semiconducting Hg_1−x_Cd_x_Te QWs of the same width, semi-metallic QWs can have much lower resistivity and lower thermal noise. For that reason, we restrict our study to the case of semi-metallic Hg_1−x_Cd_x_Te heterostructures and temperature T = 77 K.

Numerical simulation of the energy spectra and wave functions was carried out in the framework of 8-band k–p Hamiltonian (Melezhik et al. 2014; Novik et al. 2005) to incorporate strong band mixing and nonparabolicity of the dispersion law. Such modeling allows one to describe the presence of semi-metallic or semiconducting states in the well. Earlier, we have shown that HgTe quantum wells have inverted bands order (which corresponds to a semi-metallic state) for well widths larger than the critical thickness of about 7 nm (Melezhik et al. 2014).

Three efficient electron scattering mechanisms are dominant in bulk Hg_1−x_Cd_x_Te at nitrogen temperature: inelastic scattering on longitudinal optical (LO) phonons, residual charged impurities (CI) scattering, and electron–hole (EH) scattering (the latter two are elastic) (Dubowski et al. 1981). To calculate the impact of these scattering mechanisms on the electron mobility in quantum well, the linearized Boltzmann transport equation (lBTE) was iteratively solved. Direct solution of lBTE allows one to accurately include the inelasticity of electron scattering, and recovers how the carrier distribution function is perturbed by the applied electric field in the channel. The obtained approximation for the perturbed distribution function allows us to calculate the electron mobility.

## Methods

Calculations of energy spectra of Hg_0.32_Cd_0.68_Te/Hg_1−x_Cd_x_Te/Hg_0.32_Cd_0.68_Te QW with the (100) growth axis were performed in the framework of the 8-band k-p Hamiltonian (Novik et al. 2005). This method and the results obtained are described in detail in Melezhik et al. (2014). Here, we only briefly outline the calculated dependencies of carrier energy spectra and intrinsic concentrations.

**Fig. 1:**
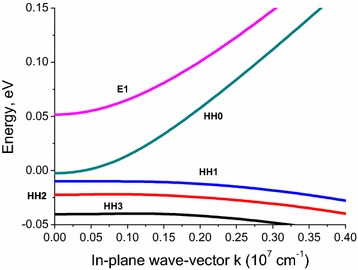
Dependence of the energy spectrum of 2D-confined carriers in Hg_0.32_Cd_0.68_Te/HgTe/Hg_0.32_Cd_0.68_Te QW with the (100) axis on the in-plane wave vector *k*. Energy levels marked HH0 to HH3 denote the ground state and the first three heavy hole levels, respectively, and E1 marks the first electron level. HH0 and E1 levels belong to the conduction band, while HH1–HH3 are levels from the valence band

The dependence of the 2D-confined carrier energy spectrum on the in-plane wave-vector *k* is plotted in Fig. 1. We have neglected the misfit strain in the spectrum calculations since it can be compensated in a multi-layered heterostructure. Therefore there is no band overlap between heavy-hole and electron states in our case.

We have also calculated the hybridized electron wave functions and incorporated them into the calculation of matrix elements for electron scattering. For the sake of simplicity, in such calculations we used the electron wave functions taken at the Fermi level.

The perturbed distribution function is obtained from the lBTE. We followed the methodology of Kawamura and Das Sarma (1992) and adapted it for the case of a nonparabolic energy dispersion law. For simplicity, we consider the case when only the ground electron level is populated, and all scattering processes take place within this level. The energy of the bottom of the ground level is denoted *E*_0_.

We consider the distribution function *f*(***r***,***k****t*) which gives the occupation probability of the state *|k*〉 by an electron in a volume element *d****r*** at the position ***r*** at time *t*. The rate of change of *f*(***r***,***k****,t*) with respect to time is given by the familiar Boltzmann equation:

1 $$\frac{\partial f}{\partial t} = - \frac{1}{\hbar }\frac{\partial E}{{\partial \varvec{k}}}\frac{\partial f}{{\partial \varvec{r}}} - \frac{1}{\hbar }\varvec{F}\frac{\partial f}{{\partial \varvec{k}}} + I_{c} \left[ f \right],$$

where *E* is the electron energy, ***F*** is the force due to the external electric field (this force for electrons is $F = - e{\text{E}}_{ext} ,$ where $E_{ext}$ is the external field, and *e* is the electron charge). The last term is the collision integral, which arises from the electron scattering and is given by:

2 $$I_{c} \left[ f \right] = - \int {\frac{{d^{2} \varvec{k}^{{\prime }} }}{{\left( {2\pi } \right)^{2} }}\left\{ {S\left( {\varvec{k},\varvec{k}^{{\prime }} } \right)f\left( {\varvec{r},\varvec{k},t} \right)\left[ {1 - f\left( {\varvec{r},\varvec{k}^{{\prime }} ,t} \right)} \right] - S\left( {\varvec{k}^{{\prime }} ,\varvec{k}} \right)f\left( {\varvec{r},\varvec{k}^{{\prime }} ,t} \right)\left[ {1 - f\left( {\varvec{r},\varvec{k},t} \right)} \right]} \right\},}$$

where *S*(***k***, ***k****′*) is the differential scattering rate from state $\left. {\left| k \right.} \right\rangle$ to state $\left. {\left| {k^{{\prime }} } \right.} \right\rangle$, bold characters are the vectors while non-bold characters are scalars. For a uniform electric field in a homogeneous system, the Boltzmann equation in the steady state becomes:

3 $$\frac{1}{\hbar }\varvec{F}\frac{{\partial f\left( \varvec{k} \right)}}{{\partial \varvec{k}}} = I_{c} \left[ f \right]$$

In equilibrium, the carrier distribution is simply given by the Fermi-Dirac occupation factor $f_{0} \left( \varvec{k} \right)$. The Fermi level for an intrinsic system is found numerically by setting the concentrations of electrons and holes in the well equal to each other. For a doped system with the given electron concentration, the Fermi level is found by fitting the electron concentration in the well to the needed value. For weak electric fields, we suppose field-induced changes of the Fermi level to be negligible.

In the presence of a weak electric field, the distribution function *f* undergoes an axially symmetric perturbation and is shifted towards the field direction. In this case *f* may be presented as

4 $$f\left( {\mathbf{k}} \right) = f_{0} \left( {\mathbf{k}} \right) - \frac{F}{\hbar }\cos \alpha \frac{\partial E}{\partial k}\frac{{\partial f_{0} }}{\partial E}\phi \left( E \right),$$

where $\alpha$ is the angle between ***k*** and ***F***, and $\phi \left( E \right)$ is the perturbation distribution, which has the physical dimension of time. One should note that the coefficients for the perturbed part of $f\left( \varvec{k} \right)$ can be written in an arbitrary form since it amounts to a rescaling of the unknown function $\phi \left( E \right)$, and the form (4) was chosen merely to simplify the further algebra. To obtain the linearized form of collision integral (2), $f\left( \varvec{k} \right)$ in the form of (4) is substituted into the expression (2), which yields the following simplified form for the collision integral:

5 $$I_{c} \left[ f \right] = \frac{F}{\hbar }\frac{{\partial f_{0} }}{\partial E}\cos \alpha \times \int {\frac{{d^{2} {\mathbf{k}}^{{\prime }} }}{{\left( {2\pi } \right)^{2} }}\frac{{1 - f_{0} \left( {E^{{\prime }} } \right)}}{{1 - f_{0} \left( E \right)}} \times \left[ {\frac{\partial E}{\partial k}\phi \left( E \right) - \cos \vartheta \frac{{\partial E^{{\prime }} }}{{\partial k^{{\prime }} }}\phi \left( {E^{{\prime }} } \right)} \right]S\left( {{\mathbf{k}},{\mathbf{k}}^{{\prime }} } \right)}$$

Substituting (4), (5) into Eq. (3) and neglecting the term proportional to F^2^ in the left-hand side of (3), one arrives at the linearized Boltzmann transport equation (lBTE):

6 $$1 = \int {\frac{{d^{2} \varvec{k}^{{\prime }} }}{{\left( {2\pi } \right)^{2} }}\frac{{1 - f_{0} \left( {E^{{\prime }} } \right)}}{{1 - f_{0} \left( E \right)}} \times \left[ {\phi \left( E \right) - cos\vartheta \frac{{\partial E^{{\prime }} /\partial k^{{\prime }} }}{\partial E/\partial k}\phi \left( {E^{{\prime }} } \right)} \right]S\left( {\varvec{k},\varvec{k}^{{\prime }} } \right),}$$

where $\vartheta$ is the angle between ***k*** and ***k****′*, while S(**k**, **k**′) is the differential scattering rate.

Consider the scattering mechanisms that are most important in MCT: the LO (longitudinal optical) phonon scattering, CI (charged impurities) scattering and EH (electron–hole) scattering. The LO phonon scattering is inelastic, while the CI and EH scattering are elastic. In HgTe, longitudinal optical phonons can be considered dispersionless, with the constant energy $\hbar w_{0} = 17\;{\text{meV}}$ (Liubchenko et al. 1984). Differential scattering rates for different scattering mechanisms are additive, thus the total differential scattering rate $S\left( {\varvec{k},\varvec{k}^{{\prime }} } \right)$ can be expressed as:

7 $$\begin{aligned} S\left( {\varvec{k},\varvec{k}^{{\prime }} } \right) & = S_{CI} \left( {\varvec{k},\varvec{k}^{{\prime }} } \right)\delta \left( {E,E^{{\prime }} } \right) + S_{EH} \left( {\varvec{k},\varvec{k}^{{\prime }} } \right)\delta \left( {E,E^{{\prime }} } \right) + S_{LO}^{a} \left( {\varvec{k},\varvec{k}^{{\prime }} } \right)\delta \left( {E + \hbar w_{0} ,E^{{\prime }} } \right) \\ & \quad + S_{LO}^{e} \left( {\varvec{k},\varvec{k}^{{\prime }} } \right)\delta \left( {E - \hbar w_{0} ,E^{{\prime }} } \right) \theta \left( {E - \hbar w_{0} - E_{0} } \right) \\ \end{aligned}$$

where $S_{LO}^{a} \left( {\varvec{k},\varvec{k}^{{\prime }} } \right)$ and $S_{LO}^{e} \left( {\varvec{k},\varvec{k}^{{\prime }} } \right)$ are the differential scattering rates for phonon absorption and emission, while $S_{CI} \left( {\varvec{k},\varvec{k}^{{\prime }} } \right)$ and $S_{EH} \left( {\varvec{k},\varvec{k}^{{\prime }} } \right)$ are the differential scattering rates for charged impurities and electron–hole scattering. Here $\theta \left( {E - \hbar w_{0} - E_{0} } \right)$ is the Heaviside (unit step) function, which reflects the fact that electrons do not scatter below the bottom of the band.

To obtain the differential scattering rate for charged impurities scattering, we have adopted the approach of Bastard (1988) for nonzero temperatures. According to Szymanska and Dietl (1978), the electron–hole scattering rate can be calculated similarly to the electron scattering rate on CI, but the concentration of charged impurities should be replaced by the effective number of holes. For the LO phonon scattering, the relevant rate for the relaxation of the carriers from the state (k_0_) is given by Mitin et al. (1999).

Electrons scatter on charged impurities, heavy holes and polar optical phonons by the electrostatic potential. Consequently, this potential is screened simultaneously by the electron and hole subsystems. Such a screening drastically reduces the effectiveness of scattering and it should be taken into account in the mobility calculations. To our knowledge, however, the explicit form of the screening function for this problem has not been established. In our calculations, we have used the two-dimensional screening function for graphene, obtained in Ref. Hwang and Das Sarma (2007). One can argue that since the energy dispersion near the Fermi level is almost linear due to k–p band mixing and strong electron gas degeneration in doped semi-metallic HgCdTe quantum wells, such a screening function should be a good approximation. Recent experiments (Brüne et al. 2014) confirm the applicability of graphene-type screening for HgTe two-dimensional systems. For charged impurities scattering and heavy-hole scattering one should use the static screening function, while for the optical phonon scattering one should use the dynamic screening function at the phonon frequency. Our modeling incorporates screening for all three considered scattering mechanisms.

Substituting (7) into (6), one can obtain:

8 $$\begin{aligned} 1 &= \phi \left( E \right)\int {\frac{{d^{2} \varvec{k}^{{\prime }} }}{{\left( {2\pi } \right)^{2} }}\left\{ {\frac{{\left[ {1 - f_{0} \left( {E + \hbar w_{0} } \right)} \right]}}{{\left[ {1 - f_{0} \left( E \right)} \right]}}S_{LO}^{a} \left( {\varvec{k},\varvec{k}^{{\prime }} } \right)\delta \left( {E + \hbar w_{0} ,E^{{\prime }} } \right)} \right.} \\ & \quad + \frac{{\left[ {1 - f_{0} \left( {E - \hbar w_{0} } \right)} \right]}}{{\left[ {1 - f_{0} \left( E \right)} \right]}}S_{LO}^{e} \left( {\varvec{k},\varvec{k}^{{\prime }} } \right)\delta \left( {E - \hbar w_{0} ,E^{{\prime }} } \right)\theta \left( {E - \hbar w_{0} - {\text{E}}_{0} } \right) \\ & \quad \left. +{ \left( {1 - cos\vartheta } \right)\left( {S_{CI} \left( {\varvec{k},\varvec{k}^{{\prime }} } \right) + S_{EH} \left( {\varvec{k},\varvec{k}^{{\prime }} } \right)} \right)\delta \left( {E,E^{{\prime }} } \right)} \right\} \\ & \quad - \phi \left( {E + \hbar w_{0} } \right)\int \frac{{d^{2} \varvec{k}^{{\prime }} }}{{\left( {2\pi } \right)^{2} }}\frac{{\left[ {1 - f_{0} \left( {E + \hbar w_{0} } \right)} \right]}}{{\left[ {1 - f_{0} \left( E \right)} \right]}} \times \left[ {cos\vartheta \frac{{\partial E^{{\prime }} /\partial k^{{\prime }} }}{\partial E/\partial k}} \right]S_{LO}^{a} \left( {\varvec{k},\varvec{k}^{{\prime }} } \right)\delta \left( {E + \hbar w_{0} ,E^{{\prime }} } \right) \\ & \quad - \phi \left( {E - \hbar w_{0} } \right)\int \frac{{d^{2} \varvec{k}^{{\prime }} }}{{\left( {2\pi } \right)^{2} }}\frac{{\left[ {1 - f_{0} \left( {E - \hbar w_{0} } \right)} \right]}}{{\left[ {1 - f_{0} \left( E \right)} \right]}} \times \left[ {cos\vartheta \frac{{\partial E^{{\prime }} /\partial k^{{\prime }} }}{\partial E/\partial k}} \right]S_{LO}^{e} \left( {\varvec{k},\varvec{k}^{{\prime }} } \right)\delta \left( {E - \hbar w_{0} ,E^{{\prime }} } \right) \\ & \quad \theta \left( {E - \hbar w_{0} - E_{0} } \right) \\ \end{aligned}$$

One should note that *δ*(*E* + ℏ*w*_0_, *E*′) and *δ*(*E* − ℏ*w*_0_, *E*′) reflect energy conservation laws that restrict the range of possible values of *k*′ in (8), since *δ*(*E*, *E*′) is equal to *δ*(*k*, *k*′), where *k* and *k*′ are scalars. Thus two-dimensional integrals over the whole 2D *k*-plane reduce to one-dimensional integrals over the angle $\vartheta$.

To obtain the perturbation function *ϕ*(*E*), we iteratively solve the Eq. (8). The iterative procedure for the calculations is described in Basu and Nag (1980). We use the modified iterative procedure (Melezhik et al. 2015). It allows us to reduce the computational time in several times in comparison with the iterative procedure from Basu and Nag (1980). In our modification of the iterative procedure, the starting values for the procedure consisting of (*n* *+* *1*) iterations were determined as follows: *ϕ*_0_(*E* − *n*ℏ*w*_0_) was found from (8) as in the standard iteration procedure (Basu and Nag 1980) (in the first step of this iterative procedure one assumes that the “upper” *ϕ*(*E* + ℏ*w*_0_) and the “lower” term *ϕ*(*E* − ℏ*w*_0_) are both equal to zero). Then *ϕ*_0_(*E* + *l*ℏ*w*_0_) (for *l* *>* *−n*) was found from (8) using *ϕ*_0_(*E* + (*l* − 1)ℏ*w*_0_) as the lower term and taking the upper term to be zero. To find n-th order term *ϕ*_*n*_(*E*) from (8) one uses $\phi_{n} \left( {E - \hbar w_{0} } \right)$as the lower term [if it is already found, if not $\phi_{n - 1} \left( {E - \hbar w_{0} } \right)$ is used and *ϕ*_*n*−1_(*E* + ℏ*w*_0_) as the upper term]. We repeat these iterations until the difference between *ϕ*_*n*−1_(*E*) and *ϕ*_*n*_(*E*) became smaller than 5 %. Such a convergence is usually reached after 3–4 iterations.

As a result of iterative solution of the lBTE, we can find the dependence of the disturbed distribution function *ϕ*(*E*). The electron mobility can be easily calculated from this function.

## Results

Under the action of the external dc electric field **E**_**ext**_, the distribution function changes from *f*_0_(*k*) to *f*(*k*) (4), and the average electron velocity in the 2DEG becomes nonzero. This causes the electric current with the density ***j***, which is found by averaging all possible electron velocities in the QW:

9 $$\varvec{j} = en\left\langle \varvec{v} \right\rangle = \frac{2}{{\left( {2\pi } \right)^{2} }}\frac{e}{\hbar }\int \frac{\partial E}{{\partial \varvec{k}}}f\left( \varvec{k} \right)d^{2} \varvec{k}$$

The drift mobility, according to its definition $\mu_{D} = j/( n_{s} F)$, can be obtained as follows:

10 $$\mu_{D} = \pi \frac{1}{{2\pi^{2} }}\frac{e}{{\hbar^{2} }}\frac{1}{{n_{s} }}\int \left( {\frac{\partial E}{{\partial \varvec{k}}}} \right)^{2} \frac{{\partial f_{0} }}{\partial E}\phi \left( E \right) \cdot k \cdot dk$$

**Fig. 2:**
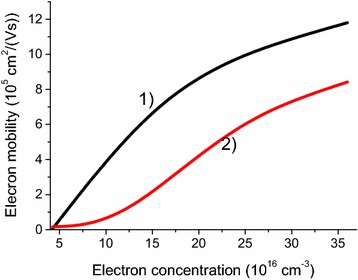
Dependence of the mobility on the electron concentration in the well. The QW width is 12 nm, the composition is Hg_0.3_Cd_0.7_Te/HgTe/Hg_0.3_Cd_0.7_Te. *Curve 1* denotes the experimental data from Tkachov et al. (2011) for T = 4.2 K. *Curve 2* shows our numerical data for T = 77 K. Concentration of residual charged impurities in the well is 1.14 × 10^15^ cm^−3^ (equal to 1.09 × 10^10^ cm^−2^)

The results of our numerical calculations for the electron mobility in HgTe quantum well of 12 nm width at T = 77 K are presented in Fig. 2. Due to the lack of experimental data on electron mobility in considered system at T = 77 K, we compared our numerical results (Fig. 2, curve 2) the experimental mobilities measured by Tkachov et al. (2011) at T = 4.2 K (Fig. 2, curve 1). In Ref. Tkachov et al. (2011), the increase of the electron concentration was achieved dynamically by applying the top-gate bias to the QW.

Comparing our numerical data to the experiment, one can see that the mobility dependencies have several similar features. First, for low electron concentrations, mobility values are fairly low in both curves. Second, such a growth of the electron concentration leads to a sharp increase of the electron mobility. These similarities could be explained by the decrease of the hole concentration and the increase of 2DEG screening while the electron concentration grows. However, experimental mobility at liquid helium temperature from Tkachov et al. (2011) increases faster than our simulated liquid nitrogen temperature mobility. This probably can be explained by a faster decrease of the hole concentration with the Fermi level shift at T = 4.2 K due to the much lower temperature. To compare quantitatively the mobility in Fig. 2 (curves 1 and 2) one should note that in the experiment, the mobility in HgTe at liquid helium temperature is 1.5–2 times larger than the mobility at liquid nitrogen temperature (Pyshkin and Ballato 2013; Meyer et al. 1992).

It is important to note that the above analysis and comparison of mobilities for different temperatures is reasonable because charged centers scattering is usually dominant in semi-metal HgCdTe structures for both liquid helium and liquid nitrogen temperatures (see the discussion below). For liquid helium temperature, this statement can be illustrated by Fig. 2 from Dubowski et al. (1981) and Fig. 5 from Meyer et al. (1992).

**Fig. 3:**
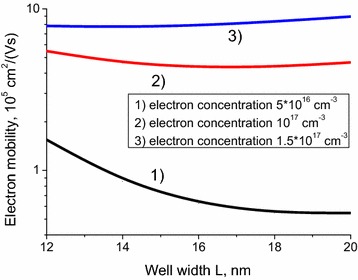
Dependence of the electron mobility on the quantum well width *L*. Curves 1, 2, 3 correspond to 5 × 10^16^, 10^17^, 1.5 × 10^17^ cm^−3^ electron concentrations in the well. The QW composition is x = 0.06. The concentration of residual charged impurities in the well is 10^15^ cm^−3^

We have also modeled the dependence of the electron mobilities in semi-metal type Hg_0.32_Cd_0.68_Te/Hg_0.94_Cd_0.06_Te/Hg_0.32_Cd_0.68_Te QW on the well width, for different electron concentrations. These results are presented in Fig. 3. The increase of mobility with the growing electron concentration can be explained by the hole concentration depletion for EH scattering mechanism and screening effect enhancement, which suppresses all three scattering mechanisms considered.

Decrease of the QW width *L* shifts the system towards the phase transition point, which is located slightly below *L* *=* 12 nm in the case under study. It is important to note that the behavior of the electron mobility as a function of the QW width is different at different electron concentrations: For high electron concentrations, the mobility does not change sufficiently, while for low electron concentration the mobility changes by factor of several times. This behavior is explained by the presence of residual charged impurities. Variation of the well width results in the variation of the hole concentration in the well (see Fig. 3). For high electron concentrations, the hole concentration is smaller than the residual impurity concentration, and the variation of holes concentration does not affect the electron mobility. For low electron concentration, the hole concentration is much greater than charged impurities concentration, and the electron mobility is strongly influenced by the change in the hole concentration.

One should note that near the band inversion point one more scattering mechanism becomes important—the scattering on fluctuations of effective mass (Tkachov et al. 2011). However, at liquid nitrogen temperature we cannot use the methodology of Tkachov et al. (2011), because due to higher temperature the simple 4-band approximation is no longer valid. Thus we can expect that near the point L = 12 nm (see Fig. 3), our simulation overestimates the electron mobility.

**Fig. 4:**
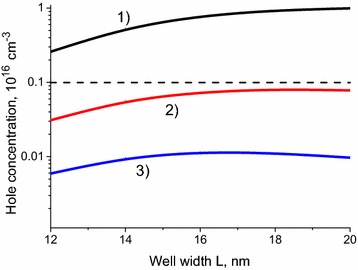
Dependence of hole concentration on the quantum well width *L*, for the same electron concentrations as presented in Fig. 3. *Curves 1, 2, 3* correspond to the electron concentrations in the well 5 × 10^16^, 10^17^, 1.5 × 10^17^ cm^−3^ respectively. The QW composition is x = 0.06. *Dashed horizontal line* represents the concentration of residual charged impurities in the well (10^15^ cm^−3^)

Consider impacts of each scattering mechanism. Strong dynamical screening leads to a strong suppression of the longitudinal optical phonon scattering. For an intrinsic 20 nm wide quantum well with the composition x  =  0, the electron mobility for the LO phonon scattering is about 3.8  ×  10^6^ cm^2^/(V s), while for n-doped quantum well of the same width with composition x  =  0.06 (electron concentration 1.5  ×  10^17^ cm^−3^), the mobility is about 6.8  ×  10^6^ cm^2^/(V s). As these mobilities are much greater than the corresponding total mobilities, we can conclude that the main contribution to the total mobility comes from the charged impurity scattering and electron–hole scattering. Relative importance of these two scattering mechanisms can be easily seen from the comparison of the hole and charged impurity concentrations presented in Fig. 4.

To use semi-metallic HgCdTe quantum well as a channel of THz hot-electron bolometer, one needs to estimate the noise in the channel. There are three main noise generation mechanisms present in this case: thermal (Johnson’s) noise, generation-recombination noise and photon noise, which can be calculated using standard formulas (Momot et al. 2010). For such an estimate one needs the bolometer channel resistance which we have extracted from the calculated values of the mobility and the channel dimensions. Further, for the estimate of the generation-recombination noise one needs to know the electron lifetime in such heterostructures. The corresponding experimental data is scarce; however, from Tkachov et al. (2011), Grein et al. (2005), Morozov et al. (2012) this time could be roughly estimated as being about 10^−10^ s.

We calculate the noises using the value of bias current of 1 mA from Dobrovolsky et al. (2010), for the quantum well of 20 nm width, with the composition x = 0.06, the electron concentration 1.5 × 10^17^ cm^−3^ and the electron mobility 9 × 10^5^ cm^2^/(V s). For those parameters the total voltage noise, which consists of the Johnson–Nyquist, generation-recombination, and photon noises, is about 3 × 10^−10^ V. The evaluated noise is almost two orders of magnitude smaller than the corresponding noise for n-type semiconductor HgCdTe bolometers from Dobrovolsky et al. (2010).

An estimate of the resistance in semi-metallic HgCdTe quantum well used as a channel of THz hot-electron bolometer shows that at high electron concentration this resistance is much smaller than 1 kΩ. This resistance is 1–2 orders of magnitude lower than the corresponding resistances in graphene channels [where the typical resistance is of the order of 10 kΩ (Du et al. 2014; Farmer et al. 2009)]. Thus, HgCdTe channels produce smaller thermal noise and, compared to graphene, can provide more efficient coupling to planar antennas in hot-electron bolometer applications.

## Conclusions

We have studied the dependence of the electron mobility in n-type semi-metallic Hg_1−x_Cd_x_Te quantum well on the well width and electron concentration. In our simulations, processes of electron scattering on longitudinal optical phonons, charged impurities, and holes have been included. Inelasticity of the LO phonon scattering have been taken into account in the iterative solution of the linearized Boltzmann transport equation. Our modeling shows that the increase of the electron concentration in the well enhances the 2D electron gas screening, decreases the hole concentration, and can ultimately lead to a high electron mobility at liquid nitrogen temperature. The increase of the electron concentration in the QW could be achieved during the QW growth by delta-doping of barriers, or dynamically by applying the top-gate bias voltage.

Our results show that for low electron concentration the increase of the QW width leads to a strong suppression of the electron mobility. At high electron concentration values, the electron mobility is much higher and depends only weakly on the QW width. These results are important in the context of establishing the optimal parameters for the fabrication of high-mobility Hg_1-x_Cd_x_Te quantum wells applications such as terahertz detectors working at liquid nitrogen temperatures.

We have also assessed advantages of the semimetal type HgCdTe QW hot-electron bolometer channel in comparison with the semiconductor HgCdTe epitaxial layer and graphene hot electron bolometers for THz applications. We conclude that HgCdTe semi-metallic QWs can demonstrate higher mobility, lower noise, higher operational speed, and can provide much more efficient coupling to planar antennas in THz range detector applications.

## References

[CR1] Bastard G (1988). Wave mechanics applied to semiconductor heterostructures.

[CR2] Basu PK, Nag BR (1980). Lattice scattering mobility of a two-dimensional electron gas in GaAs. Phys Rev B.

[CR3] Brüne C, Thienel C, Stuiber M, Böttcher J, Buhmann H, Novik EG, Liu C-X, Hankiewicz EM, Molenkamp LW (2014). Dirac-screening stabilized surface-state transport in a topological insulator. Phys Rev X.

[CR4] Dobrovolsky V, Sizov F, Zabudsky V, Momot N (2010). Mm/sub-mm bolometer based on electron heating in narrow-gap semiconductor. Terahertz Sci Technol.

[CR5] Du X, Prober DE, Vora H, Mckitterick CB (2014). Graphene-based bolometers. Graphene 2D Mater.

[CR6] Dubowski JJ, Dietl T, Szymanska W, Gakazka RR (1981). Electron scattering in CdHgTe. J Phys Chem Solids.

[CR7] Farmer DB, Chiu H-Y, Lin Y-M, Jenkins KA, Xia F, Avouris P (2009). Utilization of a buffered dielectric to achieve high field-effect carrier mobility in graphene transistors. Nano Lett.

[CR8] Grein CH, Jung H, Singh R, Flatte ME (2005). Comparison of normal and inverted band structure HgTe/CdTe superlattices for very long wavelength infrared detectors. J Electron Mater.

[CR9] Hwang EH, DasSarma S (2007). Dielectric function, screening, and plasmons in 2D graphene. Phys Rev B.

[CR10] Kawamura T, Das Sarma S (1992). Phonon-scattering-limited electron mobilities in AlGaAs/GaAs heterojunctions. Phys Rev B.

[CR11] Liubchenko AV, Salkov EA, Sizov FF (1984). Physical bases of semiconductor infrared photoelectronics (In Russian).

[CR12] Melezhik EO, Gumenjuk-Sichevska JV, Dvoretskii SA (2014). Intrinsic concentration dependences in the HgCdTe quantum well in the range of the insulator-semimetal topological transition. Semicond Phys Quantum Electron Optoelectron.

[CR13] Melezhik EO, Gumenjuk-Sichevska JV, Sizov FF (2015). Modeling of electron energy spectra and mobilities in semi-metallic Hg_1−x_Cd_x_Te quantum wells. J Appl Phys.

[CR14] Meyer JR, Arnold DJ, Hoffman CA, Bartoli FJ (1992). Electron and hole in-plane mobilities in HgTe–CdTe superlattices. Phys Rev B.

[CR15] Mitin V, Kochelap A, Stroscio A (1999). Quantum heterostructures: microelectronics and optoelectronics.

[CR16] Momot N, Zabudsky V, Tsybrii Z, Apats’ka M, Smoliy M, Dmytruk N (2010). Zero bias terahertz and subterahertz detector operating at room temperature. Semicond Phys Quantum Electron Optoelectron.

[CR17] Morozov S, Joludev M, Antonov A, Rumyantsev V, Gavrilenko V, Aleshkin V, Dubinov A, Mikhailov N, Dvoretskiy S, Drachenko O, Winnerl S, Schneider H, Helm M (2012). Study of lifetimes and photoconductivity relaxation in heterostructures with Hg_x_Cd_1−x_Te/Cd_y_Hg_1−y_Te quantum wells. Semiconductors.

[CR18] Novik EG, Pfeuffer-Jeschke A, Jungwirth T, Latussek V, Becker CR, Landwehr G, Buhmann H, Molenkamp LW (2005). Band structure of semimagnetic Hg_1−y_Mn_y_Te quantum wells. Phys Rev B.

[CR19] Olshanetsky EB, Kvon ZD, Gerasimenko Ya A, Prudkoglyad VA, Pudalov VM, Mikhailov NN, Dvoretsky SA (2014). Metal-insulator transition in a HgTe quantum well under hydrostatic pressure. JETP Lett.

[CR20] Pyshkin L, Ballato JM (2013). Optoelectronics—advanced materials and devices.

[CR21] Szymanska W, Dietl T (1978). Electron scattering and transport phenomena in small-gap zinc-blende semiconductors. J Phys Chem Solids.

[CR22] Tkachov G, Thienel C, Pinneker V, Buttner B, Brune C, Buhmann H, Molenkamp LW, Hankiewicz EM (2011). Backscattering of Dirac fermions in HgTe quantum wells with a finite gap. Phys Rev Lett.

